# Waterpipe smoking in students: Prevalence, risk factors, symptoms of addiction, and smoke intake. Evidence from one British university

**DOI:** 10.1186/1471-2458-8-174

**Published:** 2008-05-22

**Authors:** Daniel Jackson, Paul Aveyard

**Affiliations:** 1 Department of Primary Care & General Practice, University of Birmingham, Birmingham, B15 2TT, UK

## Abstract

**Background:**

Anecdotal reports suggest waterpipe smoking is becoming common in students in western countries. The aim was to examine prevalence, risk factors, symptoms of addiction, and smoke intake.

**Methods:**

This was a cross-sectional survey of students with subsidiary survey of regular waterpipe user and survey of exhaled carbon monoxide (CO) before and after waterpipe smoking in customers of a waterpipe café. 937 students of Birmingham University completed the initial survey with a follow up of 21 regular waterpipe smokers. 63 customers of a waterpipe café near the University completed the study of CO intake.

**Results:**

355 (37.9%, 95% confidence intervals (CI) 34.8 to 41.1%) students had tried waterpipes, the prevalence of trying rising with duration at University. 75 (8.0%, 95%CI 6.4 to 10.0%) were regular smokers, similar to the prevalence of cigarette smoking (9.4%). Although cigarette smoking was the major risk factor for being a regular waterpipe smoker, odds ratio (95%CI) 2.77 (1.52 to 5.06), 65% of waterpipe smokers did not smoke cigarettes. Seven of 21 (33.3%) regular waterpipe smokers experienced cravings. Nearly all regular waterpipe users thought it less harmful than smoking cigarettes. The mean (standard deviation) rise in CO was 37.4 (25.8)ppm, nearly twice as high as a typical cigarette smoker seeking cessation treatment.

**Conclusion:**

Waterpipe smoking is a common part of student culture in one British university, as in the Middle East and in the United States. It poses a potential threat to public health, with evidence of dependence and high smoke intake.

## Background

Waterpipe smoking is common among young people in Middle Eastern countries, with prevalence estimates of regular smoking of 11%–32% [1-5], and evidence of a recent increase in prevalence [6,7]. Regardless of age, most Syrian smokers began in the 1990s [8].

Data on the prevalence of waterpipe smoking in western societies is limited. A survey of American military at the turn of the century showed only 0.3% had used waterpipes in the last year [9], while a recent survey of two universities found 15%–20% had smoked a waterpipe in the past month [10]. A convenience sample of regular waterpipe users revealed the majority were young and university educated [11].

Studies with a smoking machine reveal that smoke intake from a waterpipe crucially depends upon puff topography [12,13]. One study of waterpipe smokers reported the CO boost ranged from 2 to 53 (~9.5% carboxyhaemoglobin) parts per million (ppm) in expired air, with a median of 13 ppm (~2% carboxyhaemoglobin) [14]. A study of blood carboxyhaemoglobin concentrations in Saudi Arabia showed a mean (standard deviation (SD)) of 10.1% (2.5%), higher than in Saudi cigarette smokers, 6.5% (2.7%) [15]. Nicotine intakes appear to be higher than from cigarette smokers [16], which may explain the emerging evidence of dependence [17,18].

Given the potential for harm from waterpipe smoking, we examined the prevalence, risk factors, intake of smoke, and markers of dependence in one British university.

## Method

Students were asked and volunteered to provide anonymous information in the following two studies with a view to possible publication of the data.

### Cross-sectional survey

In the cross-sectional survey, we randomly selected courses, with probability proportional to size of the course. We distributed a questionnaire in a lecture to the whole year, repeating the process for each of the three years of university. We had resources to study about 1000 students. If the prevalence of waterpipe smoking was about 5%, then we could estimate this with a precision of +/-1.4%. The survey assessed the ever use and regular use of waterpipes and other tobacco products and basic demographic information.

We asked students to leave a contact for a follow up study. We only contacted students that currently smoked at least monthly. There were 75 of these; of which 24 left contact details and 21 completed the survey. There were no major differences between the 21 regular waterpipe smokers who participated and the 54 who did not. This survey asked students for more details of use of waterpipes, symptoms of addiction, quit attempts, the social acceptability of smoking waterpipes, and perceptions of health effects.

### CO monitoring in café users

We had permission from the proprietor to monitor waterpipe smokers in a café near the University of Birmingham. Most customers were students. We recorded basic demographic data and measured expired CO immediately before and after smoking using a Micro Medical monitor. We timed the smoking session.

### Analysis

We calculated the prevalence of ever and regular use of waterpipes, defined as monthly or more frequent smoking [17,19,20]. We examined whether the demographic characteristics were risk factors for having tried waterpipe smoking in a univariate analysis and adjusted for all other factors. We also examined factors that predicted regular use among those who had tried. We calculated proportions, odds ratios (OR), and 95% confidence intervals (95%CI), using logistic regression in SPSS v14.0. For the CO data, we calculated means and SDs for the rise in CO unadjusted and adjusted for other demographic variables using linear regression.

## Results

### Cross-sectional survey

937 people completed the cross-sectional survey, and the mean (SD) age was 20.2 (2.5) years. Overall, 149 (15.9%) were current tobacco users, of which 45 (30.2%) were exclusive waterpipe smokers, 51 (34.2%) were exclusive cigarette smokers, 3 (2.0%) were exclusive cigar smokers, and 9 (6.0%) exclusive tobacco chewers. The remainder, 41 (27.5%), used more than one form of tobacco, and in 30 this included waterpipes. The prevalence (95%CI) of current waterpipe use was 8.0% (6.4–10.0%) (75 students), which was slightly less than the prevalence of cigarette smoking at 9.4% (88). Of all 75 regular waterpipe smokers, 39 (52.0%, 4.2% (3.0–5.7%) of all students) smoked weekly or more frequently, with 16 (21.3%, 1.7% (1.0–2.8%) of all students) smoking daily. Cigarette smokers smoked a mean (SD) of 7.7 (11.0) cigarettes/day.

There were 355 (37.9%, 95%CI 34.8–41.1%) students who had tried waterpipes, of whom 75 (21.1%) smoked regularly (at least monthly). The majority, 298 (83.9%) were introduced by a friend and 21 (5.9%) by family. Males were more likely than females, and those in the second year and third year were more likely to have tried. Four fifths of Arabic students had tried waterpipes, twice as many as in other ethnic groups. Males, those in higher years, Arabic students, and cigarette smokers were more likely to be regular users (Table 1). There was no evidence that gender modified the effect of ethnic group on having ever smoked waterpipes or being a regular user (χ^2 ^= 6.65, df = 5, p = 0.25 and χ^2 ^= 7.81, df = 5, p = 0.17 respectively).

**Table 1:** Prevalence of having tried and being a regular waterpipe user by demographic characteristics and cigarette use

		Ever use (n = 937 all students)			Regular use (n = 355 all who tried waterpipes)		
			
	Totals	n (%)	Unadjusted OR (95%CI)	Adjusted OR (95%CI)^2^	n (%)	Unadjusted OR (95%CI)	Adjusted OR (95%CI)^2^
Age		N/A	1.02 (0.97–1.07)^1^	1.00 (0.94–1.06)	N/A	0.91 (0.77–1.07)	0.90 (0.74–1.10)
Gender							
Female	544 (58.1)	193 (35.5%)	1.00	1.00	32 (16.6%)	1.00	1.00
Male	393 (41.9)	162 (41.2%)	1.28 (0.98–1.67)	1.49 (1.10–2.01)	43 (26.5%)	1.82 (1.09–3.04)	1.86 (1.07–3.23)
Ethnic group							
White	675 (72.0)	257 (38.1%)	1.00	1.00	48 (18.7%)	1.00	1.00
Asian	159 (17.0)	65 (40.9%)	1.13 (0.79–1.60)	1.21 (0.83–1.75)	16 (24.6%)	1.42 (0.75–2.71)	1.65 (0.85–3.23)
Chinese	36 (3.8)	5 (13.9%)	0.26 (0.10–0.68)	0.21 (0.08–0.56)	2 (40.0%)	2.90 (0.47–17.85)	2.51 (0.38–16.82)
Black	46 (4.9)	12 (26.1%)	0.57 (0.29–1.13)	0.61 (0.30–1.24)	3 (25.0%)	1.45 (0.38–5.56)	1.32 (0.33–5.36)
Arab	16 (1.7)	13 (81.3%)	7.05 (1.99–24.97)	6.44 (1.73–23.94)	5 (38.5%)	2.72 (0.85–8.69)	2.51 (0.74–8.51)
Other	5 (0.5)	3 (60.0%)	2.44 (0.41–14.70)	2.38 (0.33–17.02)	1 (33.3%)	2.18 (0.19–24.50)	1.80 (0.12–26.90)
Year of course							
1	350 (37.4)	101 (28.9%)	1.00	1.00	23 (22.8%)	1.00	1.00
2	372 (39.7)	158 (42.5%)	1.82 (1.34–2.48)	2.26 (1.61–3.18)	31 (19.6%)	0.83 (0.45–1.52)	1.25 (0.63–2.47)
3	215 (22.9)	96 (44.7%)	1.99 (1.40–2.84)	2.63 (1.74–3.96)	21 (21.9%)	0.95 (0.49–1.86)	1.79 (0.78–4.09)
Cig smoking							
No	849 (90.6)	286 (33.7%)	1.00	1.00	49 (17.1%)	1.00	1.00
Yes	88 (9.4)	69 (78.4%)	7.15 (4.22–12.11)	8.06 (4.63–14.01)	26 (37.7%)	2.93 (1.64–5.20)	2.77 (1.52–5.06)

1 For a 1 year increase in age.

2 Adjusted for all variables listed in table

Twenty one of the 75 regular waterpipe smokers completed a more detailed questionnaire. Of these, 17 (81.0%) intended to carry on smoking after the ban on smoking in enclosed public spaces was introduced (which was imminent at that time) and 15 (71.4%) smoked waterpipes at home. All but one smoker felt waterpipe smoking was socially acceptable. Nineteen (90.5%) thought waterpipe smoking was bad for health, but of these, 13 (68.4%) thought waterpipes were less damaging than cigarettes. Of the 21, two (9.5%) thought smoking waterpipes more damaging than smoking cigarettes. Two (9.5%) regular waterpipe smokers had tried to stop smoking waterpipes but restarted. Seven (33.3%) had experienced cravings to smoke waterpipes.

### CO monitoring in café users

Sixty three people smoking in waterpipe cafés participated in a survey of expired CO before and after smoking. The mean (SD) age was 22.8 (4.3) years and 14 (21.9%) were female, and they smoked a mean (SD) of 1.4 (1.2) times per week. The mean (SD) pre-smoking CO concentration was 5.1 (9.3) ppm. Seven (10.9%) had levels greater than 9 ppm (indicating current smoking), with two with high readings of 37 and 65 ppm (both regular waterpipe smokers). The sessions lasted between 15 and 60 minutes, with a mean (SD) of 29.6 (10.1) minutes. The mean (SD) reading after waterpipe smoking was 37.4 (25.8) ppm and the mean rise was 31.6 (24.9) ppm

The biggest influence on rise in CO was duration of smoking. For every 10 minutes smoking, CO rose by 15.8 (95%CI 11.0–20.6) ppm, explaining 41.6% of the variance (Table 2). There was some evidence that the rise differed by gender, age, and ethnic group, but these differences were explained by differences in session duration.

**Table 2:** Effects of characteristics of waterpipe smokers and duration of smoking on rise in exhaled carbon monoxide concentration

Characteristic	N (%)	Unadjusted coefficient (95%CI) for ppm rise in CO	Adjusted^2 ^coefficient (95%CI) for ppm rise in CO
Duration of session	29.6 (10.1)^1^	15.8 (11.0–20.6)^3^	14.8 (9.3–20.3)^3^
Age of participant in years	22.8 (4.3)^1^	1.8 (0.4–3.2)^4^	-0.1 (-1.4–1.2)^4^
Ethnic group			
White and others	43 (68.3)	Reference	Reference
Asian	28 (44.4)	-15.5 (-29.4–1.7)	-5.6 (-18.4–7.2)
Arab	15 (23.8)	3.7 (-12.5–19.9)	-1.9 (-15.3–11.5)
Gender			
Male	49 (77.8)	15.9 (5.1–26.6)	6.4 (-6.9–19.6)
Female	14 (22.2)	Reference	Reference

^1 ^Mean (SD).

^2 ^Adjusted for all variables in table.

^3 ^For a 10 minute increase in duration.

^4 ^For a 1 year increase in age.

## Discussion

This study shows that more than a third of students at one British University have tried waterpipes and of this third, one fifth are regular (at least monthly) users. Regular use of waterpipes was the second most common form of regular tobacco consumption. Most students who smoked waterpipes regularly did not smoke cigarettes. Most regular waterpipe smokers smoked at home. The factors associated with trying waterpipe smoking were similar to those associated with becoming a regular current user. Waterpipe smoking was much more common in males. The likelihood of using waterpipes was similar in all ethnic groups, except those of Arabic ethnic origin, where it was very common. A third of regular smokers showed one symptom of dependence, but this was not assessed further.

The prevalence of trying and regular waterpipe smoking increased with time in the university, which might indicate that waterpipe smoking is propagated through student culture. This is unlike cigarettes, where the prevalence of regular smoking was highest in first year students, though differences between years were small and not statistically significant. The prevalence of current waterpipe use at the only two American universities surveyed was high [10].

The students in this study thought that smoking waterpipes was relatively benign unlike students in Syria [21], but in common with American students [19,22]. It may be this perception of the relative lack of harm that supports the rise of waterpipe smoking in western students. There are no trials of interventions to deter or assist cessation of waterpipe smoking in the Cochrane review [23]. It is possible that presenting data on the known health effects could deter students from using waterpipes.

Our data show high intakes of smoke in typical waterpipe café users in the UK, with exhaled CO levels higher than observed in typical patients seeking help for stopping cigarettes [24,25]. Some of this rise might come from inhaling ambient air. Data from elsewhere suggests high intakes of nicotine from waterpipes [26], which could lead to dependence, and high intakes of other tobacco smoke constituents [27,28] causing diseases associated with smoking.

This study has some limitations. It was a cross-sectional study in one university and it is likely that the popularity of new fashions may vary. Some associations we described as risk factors may not be causal. The mean age of café users was higher than the students and the ethnic profile differed with proportionately more Arabic and Asian café users than students.

## Conclusion

These data show that waterpipe smoking is established in UK students, as in the US and we need to monitor the prevalence. It may harm public health and merits further research and a policy response.

## Competing interests

Daniel Jackson, none.

Paul Aveyard. Paul has received hospitality from various pharmaceutical companies, including those making products for smoking cessation. He has received consultancy income to him and his institution from Xenova Biotechnology, Pfizer, and McNeil AB for advice on smoking cessation.

## Authors' contributions

DJ initiated the study and it was planned by both authors. DJ carried out the study and both he and PA analysed the data. DJ wrote the first draft and PA amended it. All authors read and approved the final manuscript.

## Pre-publication history

The pre-publication history for this paper can be accessed here:


